# Linking ecosystems to public health based on combination of social and ecological systems

**DOI:** 10.1038/s41598-024-60814-z

**Published:** 2024-04-30

**Authors:** Azam Khosravi Mashizi, Mohsen Sharafatmandrad

**Affiliations:** Department of Ecological Engineering, Faculty of Natural Resources, University of Jiroft, 8th Km of Jiroft - Bandar Abbas Road, P.O. Box: 7867161167, Jiroft, Iran

**Keywords:** Biodiversity, Forest, Indicator, Rangeland, Vegetation, Health policy, Public health, Environmental impact, Ecology

## Abstract

Promotion of public health is one of the most important benefits of ecosystems. Nevertheless, the relationship between ecosystems and social health’ needs is not well understood. Therefore, a study was done to investigate the potential of natural (forests and rangelands) and artificial (urban parks and gardens) ecosystems in ensuring the five dimensions of public health (i.e. physical, mental, spiritual, social and environmental) in the social systems (urban and rural societies). Therefore, 47 health indicators were used in order to relate different ecosystems and social’ needs to five dimensions of public health through questionnaire. The results indicated that natural ecosystems had the greatest potential in providing mental, spiritual and environmental health due to ecological characteristics of wilderness and aesthetic. The artificial ecosystems had the greatest potential in providing physical and social health due to their easy access. However, there was a match between social health’ needs and ecosystem potential in the rural areas. The study highlighted the need for promotion of ecological indicators related to mental health in urban areas by enhancing silence and aesthetic in artificial ecosystems. Presented framework can provide comprehensive information on the weaknesses and strengths of different ecosystems to promote public health based on social needs and fixing the weaknesses of artificial ecosystems in urban areas.

## Introduction

Ecosystems play a very important role in providing social services to public^1,2^. One of the most important benefits of ecosystems is public health promotion^3^. Such ecosystems include gardens, urban parks, rangelands, and forests covered with trees, shrubs, and grasses^4^, acting as a health clinic promoting public health^5^. Ecosystems actually treat humans, as a functional component of ecosystems^6^. Nature provides an opportunity to restore the human psyche because human interaction with the natural environment has features that are less common in interaction with the other environments^7^ and to improve human quality of life^8^.

In fact, there is robust evidence that exposure to natural outdoor environments benefits mood^9^ and makes people feel good^7^. Health does not only mean the absence of diseases or infirmity, but also physical, spiritual, mental and social well-being^7^. Stress, depression, and family and social anomalies are common problems, increase public health expenditures in recent years. Although human health has often been dealt with in terms of physical, mental, and social dimensions^10^, little is known about the relationship between ecosystems and social^11^ and spiritual health^12^. As natural environments have a lower level of stressful architectures than man made environments, they take humans away from daily chores and force them to discover and improve their spiritual health^13^. Some believe that indicators of spirituality go beyond simple material existence including the sense of being human and the supremacy of the connection of nature or divinity (and values) such as love, compassion, and justice^14^. The results of studies revealed the importance of nature for people's health; physical activities improve mental health by improving behavior and improve social health by improving social relationships^15^. However, there are studies that did not find a significant relationship between nature and human health^16^.

In recent years, urban growth has increased with the increase in the world population^17^. Evidence shows that human impacts on ecosystems are growing^18^. Ecosystem degradation threatens public health in the future^19^. Constant exposure to artificial environments leads to fatigue, decreased vitality and health^20^. Many urban forests are shrinking and being replaced by parks^21^, so that lack of access to nature has become a serious concern worldwide^22^.

Ecosystem management faces to important challenges: whether man-made green spaces can provide all aspects of human health? and to what extent they can be considered as an alternative to natural ecosystems^23^. There is still a very important gap in scientific research regarding the health benefits of natural and artificial ecosystems^24^. It is very difficult to study the benefits of natural and artificial environments due to their complex ecological characteristics^25,26^. It is not yet clear what ecological factors are important for maximizing health benefits of the environment^25^. Understanding people's perceptions of ecosystems is one way to understand the importance of natural and artificial ecosystems for human health^27^. People have different theories about natural and artificial ecosystems^28^. Distinctive ecological indicators of natural and artificial ecosystems encourage people to choose them to visit^29^.

The distinctive indicators of ecosystems are related to the composing elements of ecosystems that discriminate an ecosystem from another one^30^. It is necessary to determine the power of the impact of important ecological indicators on health in order to determine the role of natural and artificial environments on public health. Past studies addressing the importance of ecosystems for human health have paid less attention to the socio-economic characteristics of individuals in social systems and the prevailing demands^3^. In order to determine the importance of ecosystems for public health, the needs of people in social systems must also be considered, because there are people with diverse socio-economic characteristics with very different health demands in the social systems^31^. Former studies are usually focused on one or two health dimensions^9,11,12^, while there are 5 different dimensions influencing public health (i.e. physical, mental, environmental, social, and spiritual). Both the health benefits of ecosystems and the health demands of social systems should be considered simultaneously in sustainable management. There is a long way to adequately quantify the relationships between health benefits of different ecosystems and social health’ needs. Filling these gaps may help the decision makers to balance the artificial and natural ecosystems based on people health’ demands. Knowing the most important ecological drivers of public health dimensions can also guide managers for improving artificial ecosystem characteristics related to public health promotion. Therefore, this study aimed to determine^1^ the most important ecological indicators in the ecosystem and the most important socio-economic indicators in social systems,^2^ the effect of the most important ecological indicators on public health in terms of physical, mental, spiritual, social and environmental health,^3^ the potential of natural and artificial ecosystems for physical, mental, spiritual, social and environmental health, and^4^ public health needs in the social systems.

## Materials and methods

### Study area

**Figure 1:**
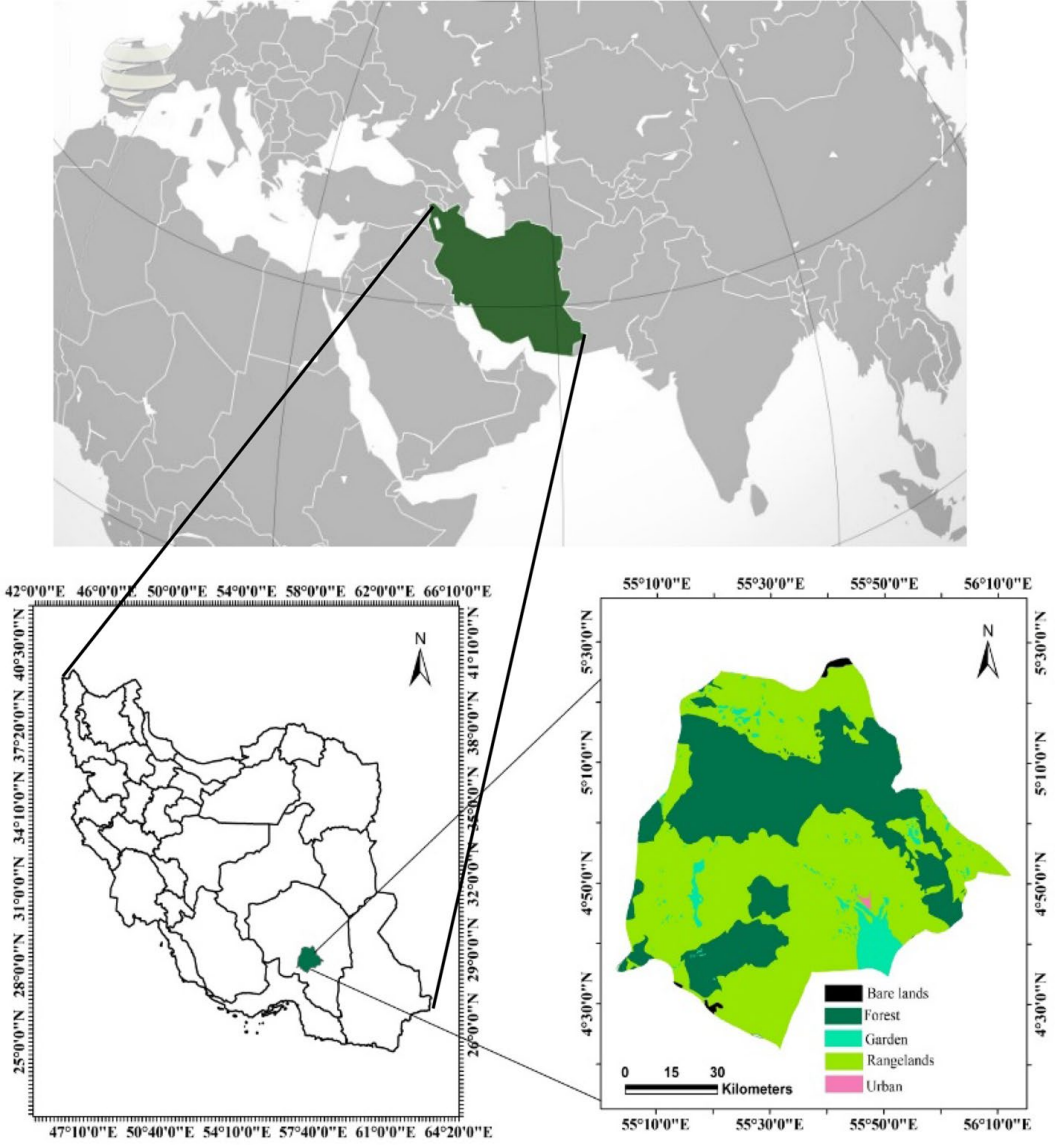
The location map of study area and its different land uses in Jiroft county, Iran. *Source*: Mapped by the authors using ArcGIS Desktop V. 10.8.

This study was conducted in Jiroft county, which is located in south east of Iran (28 40 13 N and 57 44 13 E). Jiroft city is located on the flood plains. The city covers an area about 522 square kilometers with mean elevation of 650 MASL and mean annual rainfall of 191 mm. The climate is dry. According to the 2011 census, the population of the city was 277,748. The city-level literacy rate is 80% and the unemployment rate is 30%; the population has doubled over the past 20 years^32^. The area under cultivation of horticultural crops is 52,000 hectares with a production rate of 739,000 tons. Citrus and date orchards are among the most important orchards in the city. There are eight parks and green spaces in the city. National Garden Park with an area of 15,000 square meters is the smallest and Shahid Daliri Park with an area of 130,000 square meters is the largest. Natural rangeland and forest ecosystems are located at 2511 m above sea level. Jiroft county includes 234 thousand hectares of forests, of which *Juniperus excelsa, Amygdalus lycioides* and *Pistacia atlantica* are the dominant forest species. *Artemisia aucheri* and *Astragalus spp.* are the dominant species of rangelands (Fig. 1).

### The impacts of ecosystems on public health

The impacts of both natural and artificial ecosystems on public health were examined in this study. Rangelands and forests of the study area were considered as natural ecosystems. Urban parks and private gardens were selected as artificial or manmade ecosystems. Psychophysical methods were used to study the impacts of ecosystems on public health. These methods rely on people's perception of the nature by emphasizing landscape features^33^. The output of these models is usually used for management planning^34^. To do so, participants were asked to rank their own preferences on a scale of 1 to 10. Score 1 denotes low value and score 10 denotes very high value^33^. Forty-six indicators were chosen to assess five public health criteria (physical, mental, spiritual, social and environmental health) (Appendix A). Twenty-eight ecological indicators were selected to assess the impacts of ecosystems on public health based on the literature (Appendix B).

In this study, 185 participants were selected by non-proportional quota sampling method, of which 60% were urban and 40% were rural. Data were collected using face-to-face interviews. The questionnaire had four separate sections. The first section included questions about the demographic characteristics (age, gender, education, place of residence, income, degree of dependence on the environment, occupation, etc.). The second section addressed the potential of different natural and artificial ecosystems for public health. Therefore, the respondents were asked to rank the potential of different natural and artificial ecosystems in terms of 46 mental, physical, environmental, spiritual and social health indicators. In the third section, the respondents were asked to rate the 28 ecological indicators based on their impacts on mental, physical, environmental, spiritual and social health. In the fourth section, the respondents were asked to rate the importance of 46 mental, physical, social, spiritual, social and environmental health indicators for their own health.

### Data analyses

Logistic regression was used to examine natural and artificial ecosystems in relation to health and ecological indicators. Logistic regression models are frequently used in ecology for exploring the most important environmental factors^35^. Logistic regression is an appropriate approach for analyzing hypotheses about the relationships between a categorical outcome variable and categorical predictor variables^36,37^. In its general form, the logistic regression model can be expressed as follow:

$${\text{log}}(\frac{{p}_{i}}{1-{p}_{i}})={\beta }_{0}+{\beta }_{1}{{X}_{i1}+{\beta }_{2}{X}_{i2}+{\beta }_{3}{X}_{i3}+\dots +{\beta }_{n}{X}_{in}+\varepsilon }_{i}$$

where pi is the mean of a binary variable, X_i_ is health indicator or ecological indicators for determining the ecosystem potential in providing health benefits. β is a vector of parameters to be estimated; and εi is the error term.

The coefficient of the model (*β*) is used as the probability ratio to interpret the relationship of each of significant factors in each model^36^. The probability ratio indicates the change rate of the dependent parameter in relation to the independent variables. *P*-values below 0.05 and 95% confidence intervals (CI) as statistically significant was considered for β in each model^38^.

Relationships between ecological indicators and public health were assessed by Non-metric multidimensional scaling (NMDS) using Bray–Curtis dissimilarity based on the PaST software (version 4.03)^39^. Spearman’s correlation coefficient was used to test associations between the number of ecological indicators^1–10^ of and physical, mental, social, spiritual and environmental health for NMDS axes to extract the most important ecological indicators. There are complicated relationships between ecological indicators. Hence, the path analysis model was used to reveal multivariate relationships between ecological drivers of public health resulted from NMDS. Path analysis is a generalization of multiple regressions that the strength and sign of directional relationships can be estimated for complicated relations with multiple dependent variables^40^. Path coefficient (ß) is the standardized slope of the regression of the dependent variable on the independent variable in the context of the other independent variables. Standardization was done to put different variables on the same scale. The influence of independent variables through both direct and indirect paths can be assessed in this method^41^. The chi-square test was performed to test the fit of the models which indicated a high goodness of fit for all five the models (0.10 ≤ X_2_ ≤ 2.00; 0.05 < *p* ≤ 1).

## Ethical approval and consent to participate

All experimental protocols were approved by Review Board of Faculty of Natural Resources, University of Jiroft, Iran. All methods were carried out in accordance with relevant guidelines and regulations. Informed consent was obtained from all participants.

## Results

The participants’ demographic characteristics are given in Table 1. 41% were young and 59% were adults. 47% had medium income. 51% had moderate social activity and only 15% were highly income dependent on ecosystems. NMDS showed that age and residency place were also important social characteristics affecting public health (*p <* 0.05, Table 1).

**Table 1:** Socio-economic characteristics of respondents and their correlation with the first two axes of NMDS.

Characteristics		Frequency	Percent	Axis 1	Axis 2
Gender	Female	83	45	− 0.123	− 0.135
	Male	102	55		
Age (year)	Young	76	41	− 0.478**	− 0.234
	Adult	109	59		
Education	Less than high school	43	23	+ 0.135	+ 0.235
	High school	37	20		
	Bachelor	56	30		
	Master or doctorate	49	27		
Annual income	Low	53	29	+ 0.123	+ 0.214
	Middle	87	47		
	High	45	24		
Land tenure	Private	79	43	-0.137	−0.125
	Public	106	57		
Duration of residence (year)	< 1	23	12	+ 0.278	+ 0.248
	1–10	43	23		
	10–30	84	45		
	30 <	35	20		
Income dependency on ecosystems	Low	83	45	+ 0.124	− 0.238
	Middle	75	40		
	High	27	15		
Residency place	Urban	112	60	− 0.489**	− 0.325*
	Rural	73	40		
Social activity	Low	42	23	+ 0.253	+ 0.237
	Middle	96	51		
	High	47	26		
Marital status	Single	87	47	− 0.127	− 0.137
	Married	98	53		
Number of family members	2	53	29	− 0.179	− 0.138
	2–4	86	46		
	4 <	46	25		

Significant correlations are shown by: *p = 0 05.

**Figure 2:**
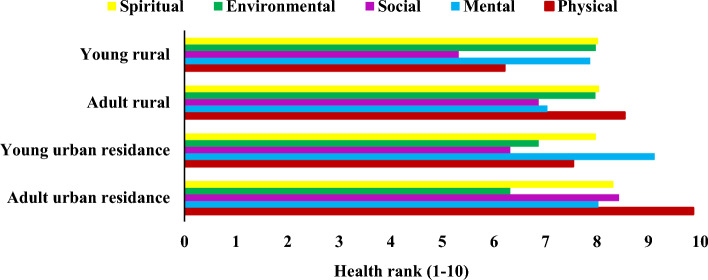
Young, adult urban and rural participants’ need in terms of physical, mental, environmental, social and spiritual health.

**Figure 3:**
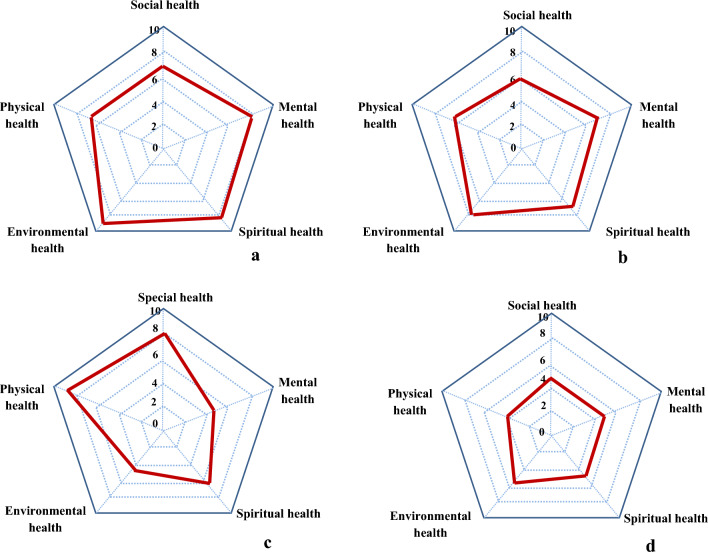
Potential of different ecosystems (**a**: forests, **b**: rangelands, **c**: parks and **d**: gardens) in providing physical, mental, social, spiritual and environmental health.

The probability ratios of health indicators in relation to different ecosystems were estimated (Table 2). Reducing feelings of anxiety and worry was important health indicator for rangeland ecosystems. The odd of rangeland important will increase 2.53 times higher when this indicator is taken into account. The most important health indicators related to forest was increase oneness with nature. The odd of forest important will increase 2.68 times higher when this indicator is taken into account. Increase social justice was the most important health indicator for park ecosystems. The odd of park important will increase 2.68 times higher when this indicator is taken into account. The most important health indicators related to garden decreased obesity. The odd of garden important will increase 1.89 times higher when this indicator is taken into account. Potential of ecosystems in providing physical, mental, social, spiritual and environmental health was shown in Fig. 2. However, forest and rangeland ecosystems were more successful in providing environmental health, park ecosystem was more successful in providing physical health. Least potential for supplying public health was belonged to garden ecosystem. Social values of physical, mental, social, spiritual and environmental health for rural and urban people were assessed (Fig. 3). Physical health was important for adults but mental health was important for younger ones.

**Table 2:** Odds ratios of the best logistic regression models for different ecosystems (rangelands, forests, parks and gardens) and health indicators.

Health indicators	Rangeland	Forest	Park	Garden
Improving endocrine and immune systems	1.12			
Reducing lung cancer			1.65	
Reducing diabetes				
Reducing respiratory diseases		1.12	1.23	1.52
Reducing higher blood pressure	1.30		1.38	
Reducing blood glucose			1.68	1.59
Decreasing Obesity	1.32		2.32	1.86
Reducing cardiovascular diseases			1.30	
Reducing infectious diseases	1.13	1.18	2.58	
Increase physical activity				
Decreasing heart rate		1.23		
Decreasing stroke			1.37	
Reducing anxiety and worry	2.53	2.11	1.28	
Reducing stress	1.35	1.44	1.86	
Reducing individual susceptibility to harm	2.36	2.19	1.13	1.16
Increasing happiness	1.56	1.39	1.32	
Decreasing fatigue	1.65	1.68	1.32	
Having good sleep	1.67	1.80		
Increasing self confidence		1.68		
Increasing life satisfaction	1.46			
Making feel better about the future	1.30	1.45		
Decreasing cognitive decline		1.30		
Feeling of love	1.39	1.89	1.30	
Increasing mental restoring capacities	1.50	2.03		
Increasing peacefulness feeling		1.23		
Increasing the tolerance threshold for adversity	1.21			
Increasing trust feeling		1.58		
Increasing feeling of concern and care for something greater than self	1.67	1.73		
Improving meditation or prayer	1.50	1.82		
Increasing beliefs relating to something beyond the human level	1.47	1.72	1.23	
Encouraging meaning and purpose in life		1.85		
Having opportunities to think on one’s life and goals	1.35			
Sense of wholeness in life		1.53		1.13
Fostering ecological commitments and activism, including biodiversity conservation	1.56	1.42	1.20	
Increasing nature reflection in one’s priorities and life	1.10	1.38		
A deep relationship with the earth	1.38	1.35		
Increasing oneness with nature	2.34	2.68		
Increasing social interaction			2.38	
Increasing social justice			2.68	
Increasing social faith				
Increasing connectedness feeling			1.31	
Increasing acceptance feeling				
A good place to spend time with family	1.59	1.23	1.35	1.28
Increasing kindness to other people			1.34	
Increasing forgiveness to other people		1.12		
Constant	− 2.13	− 3.12	− 2.16	− 3.15
AIC	1.35	2.38	3.28	2.67

Each column represents model predictors for each ecosystem. Empty cells indicate that ecosystem is not included in the best model.

The probability ratios of ecological indicators in relation to each of the natural and artificial ecosystems were estimated (Table 3). Wilderness and trees were the most important ecological indicators related to rangeland and forest ecosystems respectively. Element harmony and tree also were the most important ecological indicators related to park and garden ecosystems respectively.

**Table 3:** Odds ratios of the best logistic regression models for four ecosystems (rangeland, forest, park and garden) and ecological indicators.

Ecological indicators	Rangeland	Forest	Park	Garden
Birds		1.86	1.14	1.36
Charismatic species	1.65	1.12		
Sacred species				
Butterflies	1.23		1.38	1.52
Tree		2.53	1.37	2.19
Flower	1.80		1.68	
Strange things, fascination	1.35	1.39		
Lawn			1.30	
Complexity	1.13	1.18		
Plant richness	1.59	1.23		
Animal richness	1.32	1.68		
Elements Harmony			2.32	
How elements are shaped and arranged in space			1.28	
Water	1.30	1.23		
Aesthetic	1.67	2.11	1.43	1.32
Water purification	1.56			
Food				1.89
Reduce heat-waves		1.68		1.46
Reduce dust storms	1.30	1.45		1.30
Reduce flood		2.03		
Medical plants	1.67			
Providing shelter		1.39	1.21	
Training opportunities	1.58	1.73		
Easy to access			1.86	
Peaceful and silent place	1.18	1.35		
Ecologically sound systems	1.34	1.46		
Wilderness	2.18	2.23		
Amount of greenery	1.23	1.38	1.45	1.26
Constant	− 2.13	− 3.12	− 2.16	− 3.15
AIC	1.35	2.38	3.28	2.67

Each column represents model predictors for each ecosystem. Empty cells indicate that the ecological characteristic was not included in the best model.

**Figure 4:**
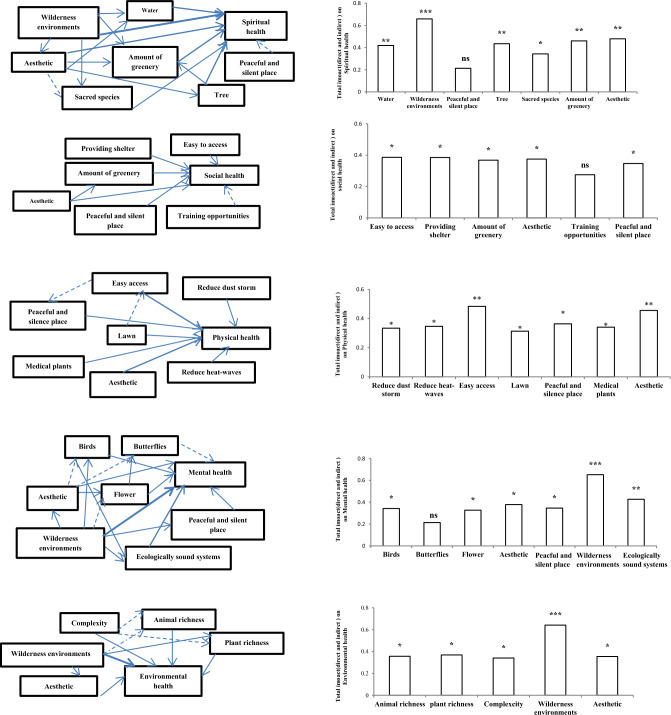
Direct, indirect and total standardized impacts on physical, mental, environmental, spiritual and social health based on Path way analysis.

**Figure 5:**
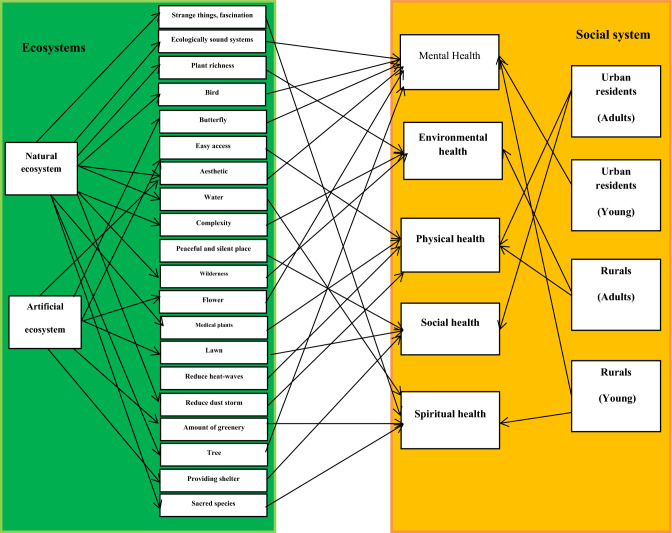
The framework for linking ecosystems to public health in social system. Arrows show significant relationships.

Results of NMDS showed that easy to access was strongly correlated with the first axis of NMDS based on physical health (*p <* 0.01, Table 4). Peaceful and silent place and aesthetic were strongly correlated with the first axis of NMDS based on mental health (*p <* 0.01). Wilderness was strongly correlated with the first axis of NMDS based on environmental health (*p <* 0.01). Aesthetic was strongly correlated with the second axis of NMDS based on spiritual health (*p <* 0.01). Providing shelter and easy to access were strongly correlated with the first axis of NMDS based on social health (*p <* 0.01, Table 4). The standardized total effect of each ecological driver on physical, mental, social, spiritual and environmental health was obtained using the direct and indirect effects of drivers (Fig. 4). Relationships between ecosystems and people health were revealed based on a framework (Fig. 5). There are natural and artificial ecosystems with different ecological characteristics which have different values for physical, mental, social, spiritual and environmental health. In social system, there are people with different demographic characteristics who need different physical, mental, social, spiritual and environmental health. This framework can help managers to identify the most important health needs in social systems and what ecological indicators should be improved to meet the needs.

**Table 4:** Correlation of ecological indicators with the two first axes of NMDS for physical, mental, social, spiritual and environmental health.

Indicators	Physical health		Mental health		Spiritual health		Environmental health		Social health	
	Axis1	Axis2	Axis1	Axis2	Axis1	Axis2	Axis1	Axis2	Axis1	Axis2
Birds	0.231	0.123	0.328*	0.213	0.146	0.128	0.106	0.145	0.265	0.216
Charismatic species	0.132	0.213	0.130	0.209	0.108	0.179	0.127	0.126	0.206	0.138
Sacred species	0.201	0.168	0.237	0.226	0.257	0.182	0.237	0.243	0.137	0.149
Butterflies	0.245	0.138	0.314*	0.136	0.108	0.239	0.139	0.201	0.219	0.124
Tree	0.238	0.213	0.209	0.242	0.348*	0.245	0.218	0.193	0.264	0.169
Flower	0.256	0.135	0.320*	0.137	0.139	0.279	0.206	0.136	0.218	0.235
Strange things fascination	0.123	0.143	0.137	0.204	0.365*	0.135	0.134	0.177	0.167	0.251
Lawn	0.205	0.339*	0.231	0.189	0.106	0.149	0.187	0.264	0.159	0.218
Complexity	0.248	0.237	0.149	0.219	0.137	0.172	0.134	0.337*	0.127	0.184
Plant richness	0.213	0.125	0.208	0.135	0.206	0.139	0.329*	0.163	0.195	0.176
Animal richness	0.238	0.139	0.217	0.240	0.184	0.128	0.343*	0.156	0.166	0.155
Elements harmony	0.128	0.237	0.184	0.167	0.137	0.187	0.204	0.208	0.218	0.147
How elements are shaped and arranged in space	0.149	0.138	0.176	0.201	0.203	0.215	0.219	0.157	0.251	0.139
Water	0.186	0.208	0.211	0.139	0.362*	0.234	0.142	0.129	0.249	0.223
Aesthetic	0.351*	0.125	0.423**	0.103	0.150	0.418**	0.199	0.336*	0.234	0.337*
Water purification	0.253	0.137	0.172	0.205	0.139	0.103	0.217	0.134	0.135	0.120
Food	0.174	0.209	0.205	0.213	0.269	0.208	0.137	0.216	0.152	0.145
Reduce heat-waves	0.230	0.342*	0.134	0.138	0.136	0.241	0.185	0.184	0.137	0.164
Reduce dust storms	0.336*	0.275	0.218	0.190	0.251	0.256	0.208	0.139	0.218	0.137
Reduce flood	0.142	0.294	0.194	0.152	0.217	0.138	0.147	0.248	0.206	0.229
Medical plants	0.319*	0137	0.126	0.137	0.106	0.194	0.136	0.105	0.213	0.148
Providing shelter	0.239	0.146	0.267	0.273	0.137	0.207	0.151	0.219	0.435**	0.162
Training opportunities	0.240	0.137	0.159	0.108	0.246	0.134	0.203	0.257	0.231	0.337*
Easy to access	0.436**	0.162	0.214	0.132	0.109	0.166	0.163	0.239	0.478**	0.143
Peaceful and silent place	0.275	0.207	0.485**	0.213	0.329*	0.205	0.204	0.136	0.137	0.154
Ecologically sound systems	0.116	0.267	0.139	0.318*	0.134	0.121	0.213	0.220	0.206	0.167
Wilderness	0.270	0.182	0.218	0.342*	0.378*	0.109	0.435**	0.139	0.249	0.219
Amount of greenery	0.237	0.137	0.108	0.143	0.314*	0.213	0.108	0.224	0.195	0.376*

Significant correlations are shown by: *p = 0 05; **p = 0.01.

## Discussion

### The most important ecological indicators and socio-economic characteristics for public health

In this study the most important ecological indicators associated with public health were identified. Knowing these indicators will help managers to identify and protect important ecosystems for public health. Wilderness was the most important ecological indicator for public health. Therefore, natural ecosystems are more important to human health than artificial ecosystems. Biodiversity (animals and plants) was one of the most important ecological indicators related to public health. The results were consistent with Fuller et al.^42^. Marselle et al.^43^ who showed that bird diversity is effective in health but plant diversity had no significant effect on health. Biodiversity also indirectly has a positive effect on public health by promoting medicinal and nutritional resources, and clean air^44,45^. In park design, natural elements are usually more valuable than artificial elements^46^. Tzoulas et al.^47^ showed that in the construction of urban parks, vegetation should be selected in a way that increases biodiversity to achieve both the goals of ecosystem sustainability and public health.

Place of residence (urban and village) and age were two important social characteristics that had the greatest impact on health. Shi et al.^48^ showed that age is an influential factor for access to green spaces for urban people. All aspects of health were more important to urban people than to rural people, which indicates the higher needs of urban health for ecosystems. Public health is at greater risk in urban areas due to substandard housing, polluted water, polluted air, congested traffic, unhealthy food, and large populations^49^. Our results indicated that physical health was important for adults and young people were more sensitive to mental health. Past results also show that mental health concerns are for younger people aged 16–24^50^ and physical health is usually important for older people^51^.

Social health was higher for the urbanites than for the villagers and higher for the urban adults than for the young. Former studies have also shown that meeting social needs and social support is essential to improving the health of older people^52^. Places with greater social cohesion usually have higher levels of health^53^. Usually the villagers are more in touch with their families and relatives and have more social interactions. So, social health is provided within the village. People who have high levels of social relationships and good relationships with their families are mentally and physically healthier^52^. Urbanites usually have more limited social relationships^54^. Therefore, they have a greater demand for social health and green space provides a good environment for their social rallies or social relations. Given that the growth rate of older people in cities is expanding^55^, paying attention to the demand of physical and social health is the most important dimension of health for urban societies. Environmental health was more important for the villagers, as they are more satisfied with their living environment, which is close to natural environments and they have more respect for nature^56^. On the other hand, the dependence of rural people on their natural environments has increased the importance of environmental health for them^2,57^.

### Interaction of natural and artificial ecosystems with the public health needs in the social systems

To determine the potential of natural and artificial ecosystems in meeting the needs of public health in the social systems, the direct and indirect effects of ecological indicators on five dimensions of health (mental, social, physical, spiritual and environmental) were identified. Easy access and aesthetic were important indicators of the environment that have the greatest impact on human physical health. A number of previous studies have shown the importance of accessible and air-conditioned green space for physical activity (such as walking, cycling)^58^.

Wilderness was the most important ecological indicator affecting mental, environmental and spiritual health. Natural forest and rangeland ecosystems were more successful in providing mental health than artificial ecosystems (parks and gardens). Franz et al.^59^ also showed that people psychologically prefer natural ecosystems to manmade ecosystems. Findings showed that stress reduction was the most important psychological indicator that affected people's mental health. Experience in natural environments not only reduces stress but can also help cure physical diseases^60^.

Scenic aesthetic is also an important factor that has a significant impact on the choice of places to visit^61,62^. The experience of beauty is determined by the combination of separate elements that the value of the elements is not the same^63^. People often judge ecosystems based on what they see^64^. People usually feel good about beautiful ecosystems^65^. Beautiful environments are not necessarily required to reduce stress, sometimes normal landscapes of green spaces in urban areas reduce stress as much as beautiful environments^66^.

People who have a lot of access to green spaces are 3.3 times more physically active than those who live in areas with minimal green spaces. They were healthier than others due to more activity^67^. According to the findings, the urban parks were the most important ecosystem for physical health. Walking, running and cycling were the most important indicators of physical health that were provided due to easy access to the parks. Previous studies have also shown a significant relationship between green space and physical activity of cycling^68^. After the parks, the forests had the greatest impact on physical health. The reduction of respiratory and heart diseases was the most important indicator of physical health provided by the forest environments, which can be attributed to the clean air of forest ecosystems^69^.

Easy access was also the most important environmental indicator for social health, and urban parks had the greatest impact on social health. In places where people feel safe and comfortable to walk, a positive perception of social cohesion is seen and interest in using green space increases^70^. Presence or access to urban green spaces increases social cohesion^71^ and are a good place for social rallies^72^.

Biodiversity can also be one of the most important reasons for choosing a place to have fun^73^. Few studies have examined the relationship between species diversity and mental health. Fuller et al.^42^ showed a positive relationship between species richness in ecosystems and the psychological benefits to human societies. Understanding biodiversity can increase the psychological well-being of human societies^74^. In this study, bird diversity was the most important indicator that affected mental health. Bird singing is often seen as a pleasant feeling^75^. Exposure to the sound of natural environments reduces stress and heart rate^76^. Birds' sounds have different effects on stress resort^77^. However, exposure to urban noises with auditory and non-auditory effects endangers human health^78^. Sound produced in cities is seen as a "waste product" that reduces human hearing^79^.

Species diversity was also the most important ecological indicator affecting environmental health because people are able to understand the distinction between species-rich and species-poor communities in ecosystems^80^. People tend to conserve biodiversity-rich ecosystems and do not feel good about changing the use of these ecosystems^81^. Biodiversity provides human access to reliable food, clean water and raw materials^82^.

Biodiversity loss has a major impact on the livelihoods of poor and vulnerable people^83^. Therefore, biodiversity is an important ecological indicator for environmental health. Aesthetic was also the most important environmental indicator affecting spiritual health and natural ecosystems played a more important role in spiritual health than artificial ecosystems. People's spiritual connection with nature has been reported in a number of previous studies^84,85^. Seeing nature inspires our superhuman strength^86^.

Among natural ecosystems, forests had a greater impact on different dimensions of health than rangelands. The two most important indicators of natural ecosystems i.e. biodiversity and aesthetic, which have the greatest impact on health, are higher in forests than in rangelands. Past studies have also shown that forests are more beautiful than rangelands^87^. The tree is a symbol of prosperity and an indicator of greenery and freshness, and a symbol of life. In arid and semi-arid areas, forests are usually more popular to visit because of the shade they provide. Forest trees have a positive effect on bird diversity by providing a good place for nesting and feeding^88^. Among the artificial ecosystems, parks were more effective than gardens in influencing public social and physical health because easy access, which was the most important indicator for artificial ecosystems in health, was provided by parks. But gardens are private property that are not open to the public.

## Conclusion

In this study, the multidimensional health benefits of ecosystems were investigated. Natural and artificial ecosystems were successful in different dimensions of health. Urban and rural people also had different health demands. However, health benefits of ecosystem and social health demands were matched in the rural areas. There was a necessary to improve the artificial ecosystems in providing mental health in the urban areas. The ecological indicators were linked to different aspects of health to help decision makers to enhance ecosystem weaknesses in providing different dimensions of health. Mental health can be improved by strengthening silence and aesthetic aspects of artificial ecosystems based on our results. In general, understanding the potential of ecosystems in meeting people's needs for different aspects of health and understanding ways to strengthen ecosystems in providing multiple health benefits help policymakers for the conservation/ development of different ecosystems.

### Supplementary Information

Supplementary Information 1.

Supplementary Information 2.

## Data Availability

The datasets used and/or analyzed during the current study are available from the corresponding author on reasonable request.
