# Supplementary material for: Linking ecosystems to public health based on combination of social and ecological systems
Source: Sci Rep. 2024 Apr 30;14:9911. doi: 10.1038/s41598-024-60814-z (PMC11061295; doi:10.1038/s41598-024-60814-z)
Supplement: Supplementary file 1 — Supplementary Information 1. [file 41598_2024_60814_MOESM1_ESM.docx]

**Appendix A. Indicators of physical, mental, spiritual, environmental and social health**

|  | **Indicators** | **Reference** |
| --- | --- | --- |
| Physical health | Improving endocrine and immune systems | Haluza et al. (2014) |
|  | Reducing lung cancer | van den Bosch and Sang (2017) |
|  | Reducing diabetes | Grellier et al. (2017); Thiering et al. (2016) |
|  | Reducing respiratory diseases | Schweitzer et al. (2018) |
|  | Reducing higher blood pressure | Markevych et al. (2014) |
|  | Reducing blood glucose | Dadvand et al. (2018) |
|  | Decreasing Obesity | Pretty (2004); Franzini et al. (2009); Markevych et al. (2016) |
|  | Reducing cardiovascular diseases | Schweitzer et al. (2018) |
|  | Reducing infectious diseases | Bonebrake et al. (2018) |
|  | Increase physical activity | McCrorie et al. (2014) |
|  | Decreasing heart rate | Cracknell et al. (2016) |
|  | Decreasing stroke | Kim et al. (2013) |
| Mental health | Reducing anxiety and worry | van den Bosch and Sang (2017); Wilson (1993) |
|  | Reducing stress | Markevych et al. (2017); Recio et al. (2016) |
|  | Reducing individual susceptibility to harm | Markevych et al. (2017); Mölter and Lindley (2015) |
|  | Increasing happiness | Cracknell et al. (2017) |
|  | Decreasing fatigue | Kaplan and Kaplan (1989) |
|  | Having good sleep | Goldberg et al. (1998) |
|  | Increasing self confidence | Goldberg et al. (1998) |
|  | Increasing life satisfaction | Ryan and Deci (2001) |
|  | Making feel better about the future | Hawks (1994) |
|  | Decreasing cognitive decline | Zunzunegui et al. (2003) |
|  | Feeling of love | Hawks (1994) |
|  | Increasing mental restoring capacities | Markevych et al. (2017); White et al. (2017) |
|  | Increasing peacefulness feeling | Wilson (1993) |
|  | Increasing the tolerance threshold for adversity | White et al. (2017) |
|  | Increasing trust feeling | Hartig et al. (2014) |
| Spiritual health | Increasing feeling of concern and care for something greater than self | Bensley (1991) |
|  | Improving meditation or prayer | Shaver et al. (2020) |
|  | Increasing beliefs relating to something beyond the human level | Bensley (1991), Fisher (2011) |
|  | Encouraging meaning and purpose in life | Shaver et al. (2020); Linton et al. (2016) |
|  | Having opportunities to think on one’s life and goals | Kaplan and Kaplan (1989) |
|  | Sense of wholeness in life | Hawks (1994) |
| Environmental health | Fostering ecological commitments and activism, including biodiversity conservation | Grim (2001) |
|  | Increasing nature reflection in one’s priorities and life | Kaplan and Kaplan (1989) |
|  | A deep relationship with the earth | Grim and Tucker (2014) |
|  | Increasing oneness with nature | Fisher (2011) |
| Social health | Increasing social interaction | Jennings and Bamkole (2019) |
|  | Increasing social justice | Rigolon et al. (2018) |
|  | Increasing social faith | Heintzman (2009) |
|  | Increasing connectedness feeling | Carpiano (2006) |
|  | Increasing acceptance feeling | Hartig et al. (2014) |
|  | A good place to spend time with family | Izenstark and Ebata (2016) |
|  | Increasing kindness to other people | Shaver et al. (2020) |
|  | Increasing forgiveness to other people | Shaver et al. (2020) |

**References**

Bensley, R.J., 1991. Defining spiritual health: a review of the literature. J Health Educ, 22(5):287–290

Bonebrake, T.C., Brown, C.J., Bell, J.D., Blanchard, J.L., Chauvenet, A., Champion, C., Chen, I.C., Clark, T.D., Colwell, R.K., Danielsen, F., 2018. Managing consequences of climate-driven species redistribution requires integration of ecology, conservation and social science. Biol Rev, 93:284–305.

Carpiano, R.M., 2006. Toward a neighborhood resource-based theory of social capital for health: Can Bourdieu and sociology help? Soc. Sci. Med., 62: 165–175.

Cracknell, D., White, M.P., Pahl, S., Depledge, M.H., 2017. A preliminary investigation into the restorative potential of public aquaria exhibits: a UK student-based study. Landsc Res, 42(1):18–32.

Cracknell, D., White, M.P., Pahl, S., Nichols, W.J., Depledge, M.H., 2016. Marine biota and psychological well-being: a preliminary examination of dose–response effects in an aquarium setting. Environ Behav, 48(10):1242–1269. https://doi.org/10.1177/0013916515597512

Dadvand, P., et al 2018. Use of green spaces and blood glucose in children; a population-based CASPIAN-V study. Environ Pollut,243(Pt B):1134–1140.

Fisher, J., 2011. The four domains model: connecting spirituality, health and well-being. Religion, 2(1):17–28.

Franzini, L., Elliott, M.N., Cuccaro, P., Schuster, M., Gilliland, M.J., Grunbaum, J.A., Franklin, F., Tortolero, S.R., 2009. Influences of physical and social neighborhood environments on children’s physical activity and obesity. Am. J. Public Health, 99: 271–278.

Goldberg, D., Oldehinkel, A.J., Ormel, J., 1998. Why GHQ threshold varies from one place to another. [Psychological Medicine](https://www.researchgate.net/journal/Psychological-Medicine-1469-8978),28: 915-21.

Grellier, J., White, M.P., Albin, M., Bell, S., Elliott, L.R., Gascón, M., Gualdi, S., Mancini, L., Nieuwenhuijsen, M.J., Sarigiannis, D.A., 2017. BlueHealth: a study programme protocol for mapping and quantifying the potential benefits to public health and well-being from Europe’s blue spaces. BMJ Open, 7:e016188

Grim, J., Tucker, M.E., 2014. Ecology and religion. Island Press, Washington, DC.

Grim, J.A., (ed) 2001. Indigenous traditions and ecology: the interbeing of cosmology and community. Harvard University Press, Cambridge, MA.

Haluza, D., Schönbauer, R., Cervinka, R., 2014. Green perspectives for public health: a narrative review on the physiological effects of experiencing outdoor nature. Int J Environ Res Public Health, 11:5445–5461

Hartig, T., Mitchell, R., de Vries, S., Frumkin, H., 2014. Nature and health. Annu Rev Public Health, 35: 207-28.

Hawks, S., 1994. Spiritual health: definition and theory. Wellness Perspect, 10(4):3–3

Heintzman, P., 2009. Nature-based recreation and spirituality: a complex relationship. Leis Sci, 32(1):72–89.

Izenstark, E., Ebata, A.T., 2016. Theorizing family-based nature activities and family functioning: The integration of attention restoration theory with a family routines and rituals perspective. Journal of Family Theory & Review, 8(2): 137-153.

Jennings, V., Bamkole, O. 2019. The Relationship between Social Cohesion and Urban Green Space: An Avenue for Health Promotion. Int J Environ Res Public Health. 16(3): 452. doi: 10.3390/ijerph16030452. PMID: 30720732; PMCID: PMC6388234.

Kaplan, R., Kaplan, S., 1989. The experience of nature: a psychological perspective. Cambridge University Press, Cambridge

Kim, E.S.; Park, N.; Peterson, C., 2013. Perceived neighborhood social cohesion and stroke. Soc. Sci. Med., 97: 49–55.

Linton, M., Dieppe, P., Medina-Lara, A., 2016. Review of 99 self-report measures for assessing wellbeing in adults: exploring dimensions of well-being and developments over time. BMJ Open, 6(7).

Markevych, I., et al 2014. A cross-sectional analysis of the effects of residential greenness on blood pressure in 10-year old children: results from the GINIplus and LISAplus studies. BMC Public Health, 14:477.

Markevych, I., Schoierer, J., Hartig, T., et al 2017. Exploring pathways linking greenspace to health: theoretical and methodological guidance. Environ Res, 158:301–317.

Markevych, I., Standl, M., Sugiri, D., Harris, C., Maier, W., Berdel, D., Heinrich, J., 2016. Residential greenness and blood lipids in children: a longitudinal analysis in GINIplus and LISAplus. Environ Res, 151:168–173.

McCrorie, P.R.W., et al 2014. Combining GPS, GIS, and accelerometry to explore the physical activity and environment relationship in children and young people – a review. Int J Behav Nutr Phys Act, 11:93.

Mölter, A., Lindley, S., 2015.Influence of walking route choice on primary school children’s exposure to air pollution–a proof of concept study using simulation. Sci Total Environ, 530:257–262

Pretty, J., 2004. How nature contributes to mental and physical health. Spirituality and Health International, 5 (2): 68-78.

Recio, A., Linares, C., Ramón Banegas, J., Díaz, J.,  2016. Road traffic noise effects on cardiovascular, respiratory, and metabolic health: an integrative model of biological mechanisms. Environ Res, 146:359–370.

Rigolon, A., Browning, M., Jennings, V., 2018. Inequities in the quality of urban park systems: An environmental justice investigation of cities in the United States. Landsc. Urban Plan., 178: 156–169.

Ryan, R.M., Deci, E.L., 2001. On happiness and human potentials: a review of research on hedonic and eudaimonic well-being. Annu Rev Psychol, 52(1):141–166.

Schweitzer, M.D., Calzadilla, A.S., Salamo, O., Sharifi, A., Kumar, N., Holt, G., Campos, M., Mirsaeidi, M., 2018. Lung health in era of climate change and dust storms. Environ Res, 163:36–42.

Shaver, N., Michaelson, V., Whitehead, R., Pickett, W., Brooks, J., 2020. Structural validity of a brief scale adapted to measure adolescent spiritual health. [SSM - Population Health](https://www.sciencedirect.com/journal/ssm-population-health), 12: 100670.

Thiering, E. et al. 2016. Associations of residential long-term air pollution exposures and satellitederived greenness with insulin resistance in German adolescents. Environ Health Perspect, 124:1291–1298. https://doi.org/10.1289/ehp.1509967

van den Bosch, M., Sang, A.O., 2017. Urban natural environments as nature-based solutions for improved public health – a systematic review of reviews. Environ Res, 158:373–384

White, M.P., Weeks, A., Hooper, T., et al 2017. Marine wildlife as an important component of coastal visits: the role of perceived biodiversity and species behaviour. Mar Policy, 78(80):89

Wilson, E.O., 1993. Biophilia and the conservation ethic. In: Kellert SR, Wilson EO (eds) The biophilia hypothesis. Island Press, Washington, DC, p 31

Zunzunegui, M.-V., Alvarado, B.E., Del Ser, T., Otero, A., 2003. Social networks, social integration, and social engagement determine cognitive decline in community-dwelling spanish older adults. J. Gerontol Ser., 58: 93–100.
