# Supplementary material for: Linking ecosystems to public health based on combination of social and ecological systems
Source: Sci Rep. 2024 Apr 30;14:9911. doi: 10.1038/s41598-024-60814-z (PMC11061295; doi:10.1038/s41598-024-60814-z)
Supplement: Supplementary file 2 — Supplementary Information 2. [file 41598_2024_60814_MOESM2_ESM.docx]

**Appendix B. Ecological criteria and their indicators related to people health**

| **Criteria** | **Indicators** | **Reference** |
| --- | --- | --- |
| Structural elements  (Kaplan and Kaplan, 1989) | Birds | Wolf et al. (2017) |
|  | Charismatic species | Dallimer et al. (2012) |
|  | Sacred species | Ntiamoa-Baidu (2008) |
|  | Butterflies | Marselle et al. (2016) |
|  | Trees | Kaplan and Kaplan (1989) |
|  | Flowers | Daniels et al. (2018) |
|  | Strange things, fascination | James (1892) |
|  | Lawn | Daniels et al. (2018) |
| Biodiversity  (Liddicoat et al., 2018) | Complexity | Kaplan and Kaplan (1989) |
|  | Plant richness | Stevens (2018) |
|  | Animal richness | White et al. (2017) |
|  | Elements harmony | Hawks (1994) |
|  | How elements are shaped and arranged in space | Ode et al. (2010) |
| Ecosystem services  (M.E.A, 2005) | Water | Schwarz et al. (2017) |
|  | Aesthetic | Schwarz et al. (2017) |
|  | Water purification | Harrison et al. (2014) |
|  | Food | Harrison et al. (2014‎) |
|  | Heat-waves reduction | Poumadere et al. (2005) |
|  | Dust storms reduction | Fisher et al. (2017); Schweitzer et al. (2017) |
|  | Flood reduction | European Environment Agency (2017) |
|  | Medical plants | Mace et al. (2012) |
|  | Shelter provision | Mace et al. (2012) |
|  | Training opportunities | Tolvanen et al. (2020) |
| Environmental condition (Egorov et al., 2017) | Easy to access | van den Berg et al. (2017) |
|  | Peaceful and silent place | Tolvanen et al. (2020) |
|  | Ecologically sound systems | Grahn and Stigsdotter (2010) |
|  | Wilderness | Gobster et al. (2007) |
|  | Amount of greenery | Cohen-Cline et al. (2015) |

**References**

Cohen-Cline, H., Turkheimer, E., Duncan, G.E., 2015. Access to green space, physical activity and mental health: a twin study. J Epidemiol Community Health, 69(6):523–529.

Dallimer, M., Irvine, K.N., Skinner, A.M.J., Davies, Z.G., Rouquette, J.R., Maltby, L.L., et al. 2012. Biodiversity and the feel-good factor:Understanding associations between self-reported human well-being and species richness. BioScience, 62: 47–55.

Daniels, B., Zaunbrecher, B.S., Paas, B., Ottermanns, R., Ziefl, M., Roß-Nickoll, M., 2018. Assessment of urban green space structures and their quality from a multidimensional perspective. Science of the Total Environment, 615: 1364–1378.

Egorov, A.I., Griffin, S.M., Converse, R.R., Styles, J.N., Sams, E.A., Wilson, A., Jackson, L.E., Wade, T.J. 2017. Vegetated land cover near residence is associated with reduced allostatic load and improved biomarkers of neuroendocrine, metabolic and immune functions. Environ Res. 158: 508-521. doi: 10.1016/j.envres.2017.07.009.

European Environment Agency, 2017. Climate change, impacts and vulnerability in Europe: an indicator based report.

Fisher, J.E., Andersen, Z.J., Loft, S., Pedersen, M., 2017. Opportunities and challenges within urban health and sustainable development. Curr Opin Environ Sustain, 25:77–83.

Gobster, P.H., Nassauer, J.I., Daniel, T.C., Fry, G., 2007. The shared landscape: what does aesthetics have to do with ecology? Landsc Ecol, 22(7):959–972.

Grahn, P., Stigsdotter, U.K., 2010. The relation between perceived sensory dimensions of urban green space and stress restoration. Landscape and Urban Planning, 94: 264-275.

Harrison, P.A., Berry, P.M., Simpson, G., Haslett, J.R., Blicharska, M., Bucur, M., Dunford, R., Egoh, B., Garcia-Llorente, M., Geamănă, N., Geertsema, W., Lommelen, E., Meiresonne, L., Turkelboom, F., 2014. Linkages between biodiversity attributes and ecosystem services: a systematic review. Ecosyst Serv, 9:191–203.

Hawks, S., 1994. Spiritual health: definition and theory. Wellness Perspect, 10(4):3–3

James, W., 1892. Psychology: the briefer course. Holt, New York.

Kaplan, R., Kaplan, S., 1989. The experience of nature: a psychological perspective. Cambridge University Press, Cambridge.

Liddicoat, C., Bi, P., Waycott, M, Glover, J, Lowe A.J., Weinstein. P. 2018. Landscape biodiversity correlates with respiratory health in Australia. J Environ Manage. 206:113-122. doi: 10.1016/j.jenvman.2017.10.007.

Mace, G.M., Norris, K., Fitter, A.H., 2012. Biodiversity and ecosystem services: a multilayered relationship. Trends Ecol Evol, 27:24–31.

Marselle, M.R. Irvine, K.N., Lorenzo-Arribas, A., [Warber](https://www.sciencedirect.com/science/article/abs/pii/S0272494416300275#!), S.L., 2016. Does perceived restorativeness mediate the effects of perceived biodiversity and perceived naturalness on emotional well-being following group walks in nature?. J Environ Phychol, 46:217–32.

M.E.A. 2005. A Report of the Millennium Ecosystem Assessment. Ecosystems and Human Well-Being. Island Press, Washington DC.

Ntiamoa-Baidu, Y., 2008. Indigenous beliefs and biodiversity conservation: the effectiveness of sacred groves, taboos and totems in Ghana for habitat and species conservation. J Stu Relig Nat Cult, 2(3):309–326.

Ode, Å., Hagerhall, C.M., Sang, N., 2010. Analysing visual landscape complexity: theory and application. Landsc Res, 35(1):111–131

Poumadere, M., Mays, C., Le Mer, S., Blong, R., 2005. The 2003 heat wave in France: dangerous climate change here and now. Risk Anal, 25(6):1483–1494.

Schwarz, N., Moretti, M., Bugalho, M.N., Davies, Z.G., Haase, D., Hack, J., Hof, A., Melero, Y., Pett, T.J., Knapp, S.,  2017. Understanding biodiversity-ecosystem service relationships in urban areas: a comprehensive literature review. Ecosyst Serv, 27:161–171

Schweitzer, M.D., Calzadilla, A.S., Salamo, O., Sharifi, A., Kumar, N., Holt, G., Campos, M., Mirsaeidi, M., 2018. Lung health in era of climate change and dust storms. Environ Res, 163:36–42.

Stevens, P., 2018. Fractal dimension links responses to a visual scene to its biodiversity. Ecopsychology. 10: 89-96.<https://doi.org/10.1089/eco.2017.0049>

Tolvanen, A., Kangas, K., Tarvainen, O., Huhta, E., Jäkäläniemi, A., Kyttä, M., Nikula, A., Nivala, V., Tuulentie, S., Tyrväinen, L., 2020. The relationship between people’s activities and values with the protection level and biodiversity. Tourism Management, 81: 104141.

van den Bosch, M., Sang, A.O., 2017. Urban natural environments as nature-based solutions for improved public health – a systematic review of reviews. Environ Res, 158:373–384

White, M.P., Weeks, A., Hooper, T., et al 2017. Marine wildlife as an important component of coastal visits: the role of perceived biodiversity and species behaviour. Mar Policy, 78(80):89

Wolf, L.J., zu Ermgassen, S., Balmford, A., et al 2017. Is variety the spice of life? An experimental investigation into the effects of species richness on self-reported mental well-being. PloS ONE, 12(1):e0170225.
